# Enhancing Pseudo-Telepathy in the Magic Square Game

**DOI:** 10.1371/journal.pone.0064694

**Published:** 2013-06-06

**Authors:** Łukasz Pawela, Piotr Gawron, Zbigniew Puchała, Jan Sładkowski

**Affiliations:** 1 Institute of Theoretical and Applied Informatics, Polish Academy of Sciences, Gliwice, Poland; 2 Institute of Physics, Jagiellonian University, Kraków, Poland; 3 Institute of Physics, University of Silesia, Katowice, Poland; University of Calgary, Canada

## Abstract

We study the possibility of reversing an action of a quantum channel. Our principal objective is to find a specific channel that reverses as accurately as possible an action of a given quantum channel. To achieve this goal we use semidefinite programming. We show the benefits of our method using the quantum pseudo-telepathy Magic Square game with noise. Our strategy is to move the pseudo-telepathy region to higher noise values. We show that it is possible to reverse the action of a noise channel using semidefinite programming.

## Introduction

Quantum game theory is an interdisciplinary field that combines game theory and quantum information. It lies at the crossroads of physics, quantum information processing, computer and natural sciences. Various quantizations of games were presented by different authors [1]–[5].

Quantum pseudo-telepathy games [6] form a subclass of quantum games. A game belongs to the pseudo-telepathy class providing that there are no winning strategies for classical players, but a winning strategy can be found if the players share a sufficient amount of entanglement. In these games quantum players can accomplish tasks that are unfeasible for their classical counterparts. It has been shown [7] that noise in a quantum channel can decrease the probability of winning the Magic Square game even below the classical threshold.

Noise is an unavoidable ingredient of a quantum system. Therefore its thorough investigation is a fundamental issue in quantum information processing. Quantum game theory has several potential applications (e.g quantum auctions [8]) that may be hindered by noise effects. Our previous investigation of quantum noise effects in quantum games [7], [9], [10], and quantum algorithms performance [11] revealed several interesting issues that act as an incentive of the present work. The tools developed in this work can be used to analyse the behaviour of quantum channels in other settings.

### Motivation

The motivation to study the Magic Square game and pseudo-telepathy games in general is that their physical implementation could provide convincing, even to a layperson, demonstration that the physical world is not local realistic. By *local* we mean that no action performed at some location X can have an effect on some remote location Y in a time shorter then that required by light to travel from X to Y. *Realistic* means that a measurement can only reveal elements of reality that are already present in the system [6].

Given a pseudo-telepathy game, one can implement a quantum winning strategy for this game [6]. In an ideal case, the experiment should involve a significant number of rounds of the game. The experiment should be continued until either the players lose a single round or the players win such a great number of rounds, that it would be nearly impossible if they were using a classical strategy.

In the particular case of the magic square game the classical strategy allows the players to achieve the success rate no larger than 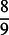. In theory, the success rate of the quantum strategy is equal to one. But any physical implementations of a quantum protocol cannot be perfect because it is subject to noise.

In particular, the players, Alice and Bob, must be so far away from each other that the time between the question and their respective answers is shorter than the time required by light to travel between their locations. This set-up involves sending parts of an entangled quantum state to two remote locations. Sending qubits through a channel will inevitably add noise to the system. Our aim is to counteract this noise. In this paper we focus on the destructive aspects of the process of transmission of a qubit through a noisy separable quantum channel and introduce a scheme that allows the partial reversion of the channel action. This reversal gives rise to the players' success rate above the classical limit of 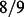 for some parameters of noisy channels. Our scheme for reversing an action of a noisy channel may prove valuable in future experimental set-ups of such games.

### Magic square game

The magic square is a 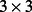 matrix filled with numbers 0 or 1 so that the sum of entries in each row is even and the sum of entries in each column is odd. Although such a matrix cannot exist (see Table 1) one can consider the following game.

**Table 1:** An illustrative filling of the magic square with numbers 0 and 1.

1	1	0
1	0	1
1	0	?

The question mark shows that it is not possible to put a number in the last field and satisfy both conditions of the game.

The game setup is as follows. There are two players: Alice and Bob. Alice is given a row, Bob is given a column. Alice has to give the entries for a row and Bob has to give entries for a column so that the parity conditions are met. Winning condition is that the players' entries at the intersection must agree. Alice and Bob can prepare a strategy but they are not allowed to communicate during the game.

There exists a (classical) strategy that guarantees the winning probability of 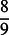. If the parties are allowed to share a quantum state they can achieve probability of success equal to one [6].

In the quantum version of this game [12], [13] Alice and Bob are allowed to share an entangled quantum state. The winning strategy is following. Alice and Bob share an entangled state:
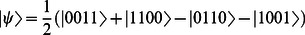
(1)


and apply local unitary operators forming operator 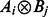, where

• 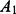  = 
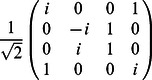
,

• 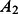  = 
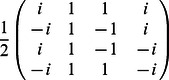
,

• 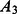  = 
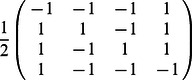
,

• 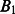 
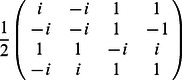
,

• 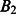 
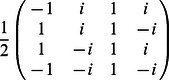
,

• 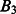  = 
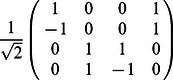
.

Indices 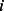 and 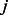 label rows and columns of the magic square. The state of this scheme before measurement is
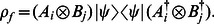
(2)


The final step of the game consists of the measurement in the computational basis.

In [7], the situation where the initial state 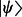 is corrupted by the noise was investigated. Therefore, Eq. 2 is transformed into
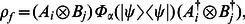
(3)where 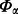 denotes one-parameter family of noisy quantum channels.

In such a case it is justified to inquire what is the mean probability of Alice and Bob's success given the amount of noise introduced by channel 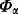. The mean probability 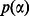 of measuring the outcome yielding success in the state 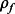 is given by
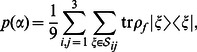
(4)where 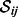 is the set of right answers for the column and row 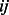 (Table 2). The mean is taken over all pairs 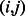.

**Table 2:** Sets 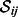 – plus sign (+) indicates that the given element belongs to the set, minus (−) sign indicates that the element does not belong to the set.

	0	1	2	3	4	5	6	7	8	9	10	11	12	13	14	15
*S*_11_	+	+	−	−	+	+	−	−	−	−	+	+	−	−	+	+
*S*_12_	+	+	−	−	−	−	+	+	+	+	−	−	−	−	+	+
*S*_13_	+	+	−	−	−	−	+	+	−	−	+	+	+	+	−	−
*S*_21_	+	−	+	−	+	−	+	−	−	+	−	+	−	+	−	+
*S*_22_	+	−	+	−	−	+	−	+	+	−	+	−	−	+	−	+
*S*_23_	+	−	+	−	−	+	−	+	−	+	−	+	+	−	+	−
*S*_31_	−	+	+	−	−	+	+	−	+	−	−	+	+	−	−	+
*S*_32_	−	+	+	−	+	−	−	+	−	+	+	−	+	−	−	+
*S*_33_	−	+	+	−	+	−	−	+	+	−	−	+	−	+	+	−

A winning strategy exists for noiseless channels. In the case of noisy channel, the same strategy gives a higher probability of winning than in the classical case for low noise channels [7]. The objective of this work is to find local channels that partially reverse the action of the noise and therefore extends the pseudo-telepathy to channels with higher noise. In order to achieve this, Eq. 3 is transformed into
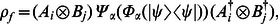
(5)where 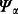 denotes local channel with respect to Alice and Bob's subsystems that allows to raise their probability of winning 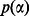. In order to achieve that a series of semi-definite optimization programs has to be numerically solved.

### Quantum channels

In the most general case, the evolution of a quantum system can be described using the notion of a *quantum channel* [14]–[16]. A quantum channel is a mapping acting on density operators 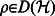, i.e., operators where 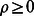 and 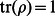 on a Hilbert space 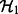 and transforming them into operators on a another Hilbert space 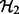. Thus
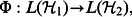
(6)where 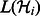 denotes the set of linear operators on 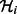. To form a proper quantum channel, the mapping 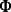 must satisfy the following restrictions:

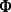
 must be *trace-preserving*, that is 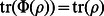,

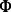
 must be *completely positive*, that is 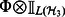 is a positive mapping, thus
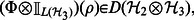
(7)for every choice of 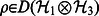 and every choice of finite-dimensional Hilbert space 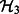, where 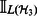 is an identity channel on the space 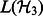.

The notion of a *product* quantum channel is introduced as follows [17]. For any choice of quantum channels that satisfy
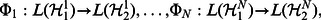
(8)we define a linear mapping
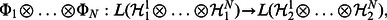
(9)to be the unique mapping that satisfies the equation
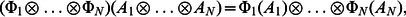
(10)for all operators 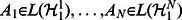.

Many different representations of quantum channels can be chosen, depending on the application. Among these are the Jamiołkowski representation, the Kraus representation and the Stinespring representation. These three representations will be used throughout this paper.

The Jamio

kowski representation of a quantum channel 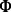 is given by
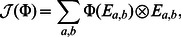
(11)where 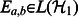 are operators with all entries equal to zero, except the entry 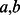 equal to one. From this definition, it is straightforward to observe that 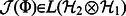. By the Choi's [14] theorem a channel is completely positive if and only if 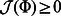. It is trace-preserving if and only if



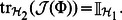
(12)Finally, the action of a quantum channel in the Jmiołkowski representation is given by
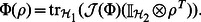
(13)


The Kraus representation of a quantum channel is given by a set of operators 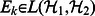. The action of quantum channel 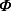 is given by:
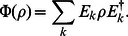
(14)


This form ensures that the quantum channel is completely positive. For it to be also trace-preserving we need to impose the following constraint on the Kraus operators
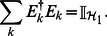
(15)


Finally, given a mapping 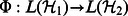 let us take another Hilbert space 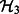 such that 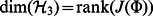 and a linear isometry 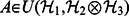. The action of a quantum channel is given by
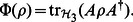
(16)


This representation is called the Stinespring representation of 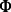.

For further discussion of quantum channels see *e.g.* [15] or [17].

### Quantum noise

In the literature, several one-parameter families of qubit noisy channels are discussed [15]. For all the families of channels listed below the parameter 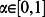 represents the amount of noise introduced by the channel. The symbols 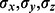 denote Pauli operators. The Kraus operators for typical noisy channels are for

• depolarising channel:

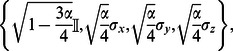

• amplitude damping: 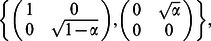

• phase damping: 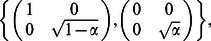

• phase flip: 
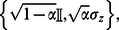

• bit flip: 
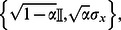

• bit-phase flip: 
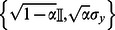
.

In order to apply noise operators to multiple qubits we form new set of operators acting on a larger Hilbert space.

Given a set of 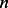 one-qubit Kraus operators 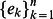 it is possible to construct new set of 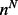 operators 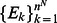 that act on a Hilbert space of dimension 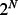 by taking Cartesian product of one-qubit Kraus operators in the following way
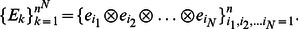
(17)


Application of the above to Kraus operators listed before gives one-parameter families of local noisy channels. This form will be used in further investigations.

## Results and Discussion

We propose the following scheme for reversing an action of a channel using semidefinite programming (SDP). In our case, the most useful formulation of a semidefinite program is as follows (after Watrous [17]).

A semidefinite program is a triple (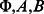) where 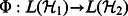 is a Hermiticity-preserving map and 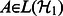 and 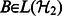 are Hermitian operators for some choice of Hilbert spaces 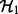 and 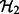. Two optimization problems are associated with the triple 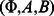, the primal and dual problems. We will focus our attention on the primal problem, which has the form:
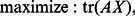


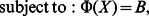


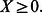



In the case of the pseudo-telepathy game, it seems appropriate to look for a channel in a product form. This is due to the fact, that Alice and Bob are separated and each of them must apply a local channel. To model this situation, let us consider the Jamio

kowski representations of Alice's and Bob's channels, denoted 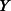 and 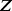 respectively. The resulting channel is given by
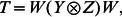
(18)where 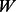 is an operator defined as follows
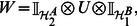
(19)where 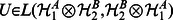 is the swap operation of subsystems 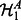 and 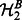, defined as
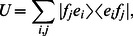
(20)for 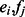 being elements of orthonormal bases of 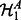 and 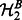 respectively.

Next, let us denote by 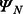 the noise channel and we put 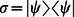. For simplicity of further calculations, let us write 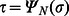 and 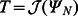. Consider the following maximization criterion problem
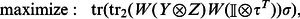
(21)which means we aim to find a channel that reverses the action of the noise channel as accurately as possible. Unfortunately, a maximization criterion in this form does not yield an SDP problem. To formulate this problem as an SDP, we first conduct some simple calculations that allow us to rewrite the maximization condition (21) as
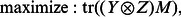
(22)

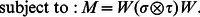
(23)


Considering the value of 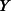 to be fixed and using the equation 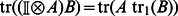, allows us to write the following SDP
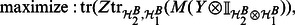


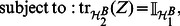
(24)

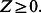



Fixing the value of 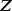 and following a similar calculation give the following SDP problem
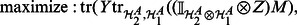


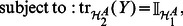
(25)

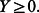



Now, we use the following algorithm to find an optimal channel. The algorithm in each iteration optimizes only a single part of the product channel. This algorithm was implemented using the SDPLR library [18], [19].

### Analysis

**Figure 1:**
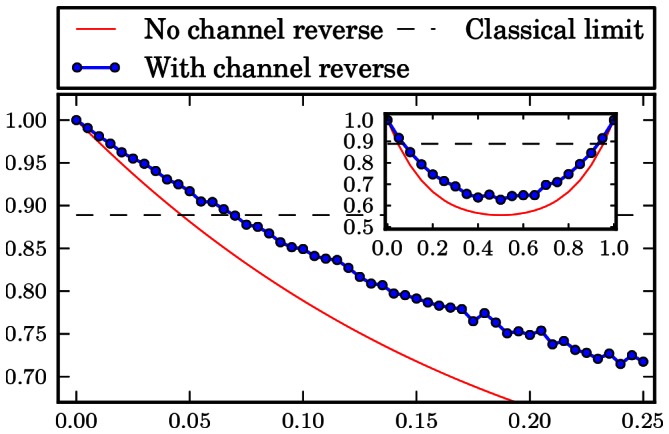
Phase flip channel. Probability of winning the pseudo-telepathy game with and without the use of our approach as a function of the noise parameter α for the phase flip channel. The inset shows the probability of winning for α∈ [0; 1].

**Figure 2:**
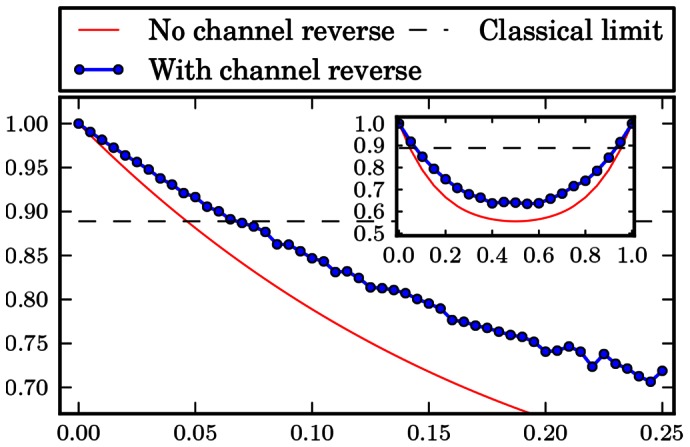
Bit flip channel. Probability of winning the pseudo-telepathy game with and without the use of our approach as a function of the noise parameter α for the bit flip channel. The inset shows the probability of winning for α∈ [0; 1].

**Figure 3:**
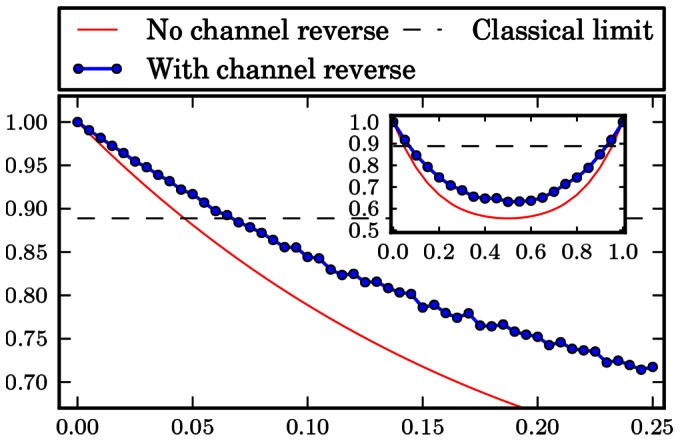
Bit phase flip channel. Probability of winning the pseudo-telepathy game with and without the use of our approach as a function of the noise parameter α for the bit phase flip channel. The inset shows the probability of winning for α∈ [0; 1].

**Figure 4:**
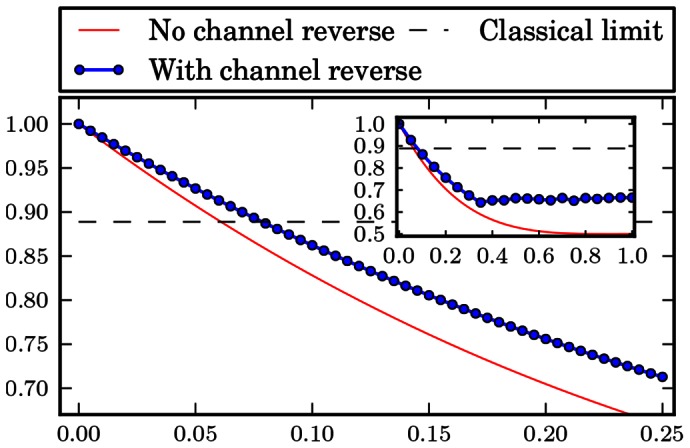
Depolarising channel. Probability of winning the pseudo-telepathy game with and without the use of our approach as a function of the noise parameter α for the depolarising channel. The inset shows the probability of winning for α∈ [0; 1].

**Figure 5:**
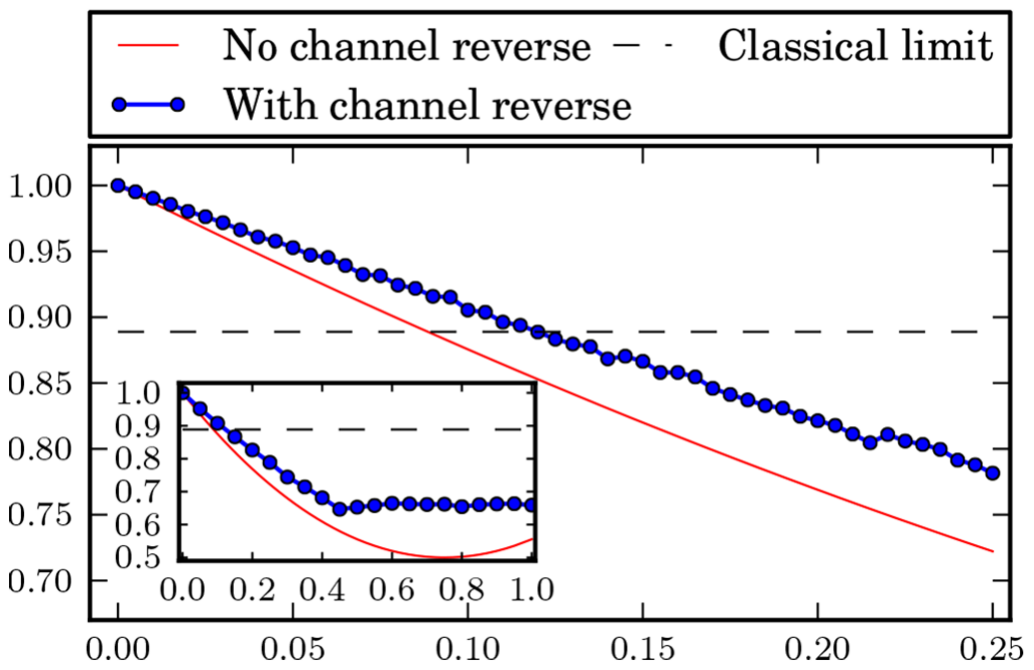
Amplitude damping channel. Probability of winning the pseudo-telepathy game with and without the use of our approach as a function of the noise parameter α for the amplitude damping channel. The inset shows the probability of winning for α∈[0; 1].

**Figure 6:**
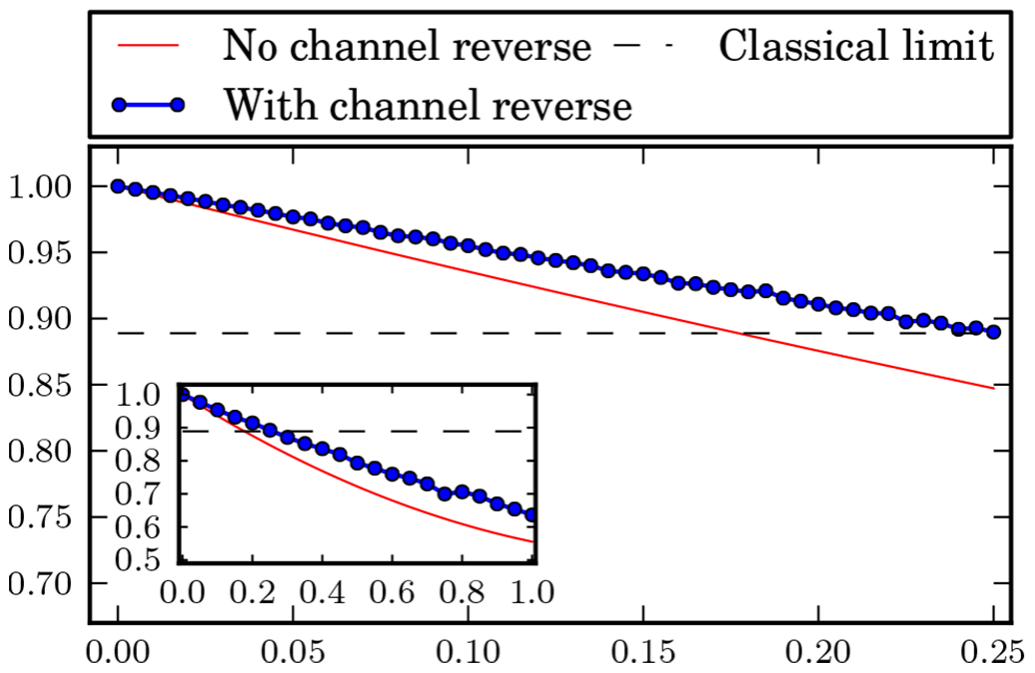
Phase damping channel. Probability of winning the pseudo-telepathy game with and without the use of our approach as a function of the noise parameter α for the phase damping channel. The inset shows the probability of winning for α∈ [0; 1].

The numerical results are gathered in form of plots at the end of the paper. Figs. 1, 2, 3, 4, 5, 6 show the results of the optimization scheme shown in Fig, 7. The application of the SDP allowed us to achieve greater winning probability for all types of noisy channels.

**Figure 7:**
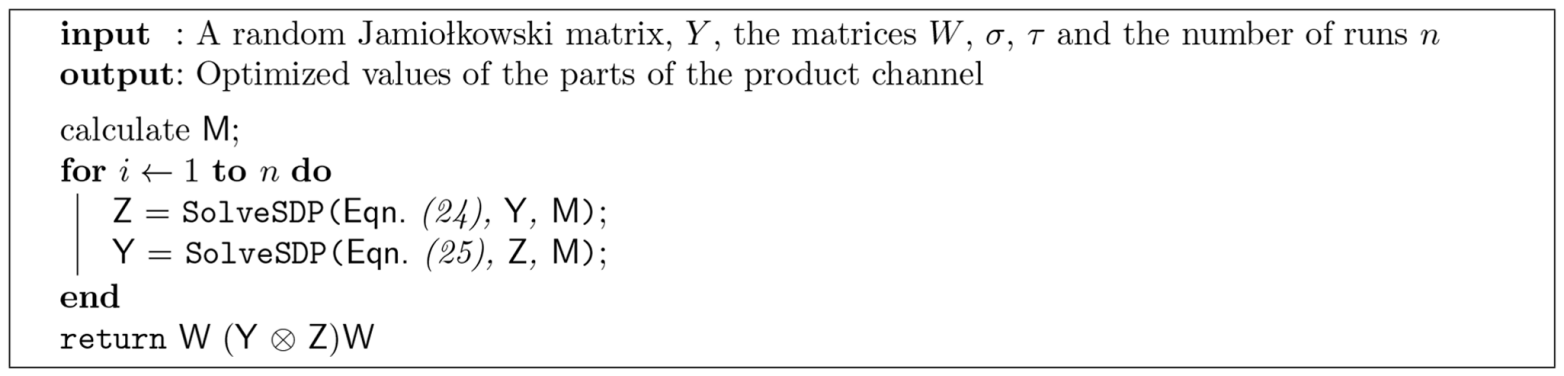
SDP optimization algorithm. SDP optimization of a product channel with a random Jamio lkowski matrix [20] as input.

In the case of the flip channels the obtained results are depicted in Figs. 1, 2 and 3. These plots show that it is possible to reverse the action of the noise channel for all values of the noise parameter 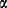. Hence, we are able to observer quantum pseudo-telepathy for higher noise channels. Furthermore, the use of our optimization method results in a plot of probability of winning as a function of the noise parameter 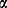 which has a shape similar to the case when we do not try to reverse the action of a channel.

Next, we move to the depolarising channel. The results obtained in this case are shown in Fig. 4. Likewise, in this case our method has also allowed us to achieve pseudo-telepathy for higher values of the noise parameter 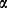. The details are depicted in the inset in Fig. 4. Additionally, for values of the noise parameter 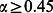 the probability of winning the game stabilizes around 0.65, opposed to the case with no channel action inverse, where it decreases to 0.5. Hence, we are able to retrieve some information in the case of high noise, local depolarising channels acting on many qubits.

Finally, we switch to the damping channels. Numerical results for this case are depicted in Figs. 5 and 6. Moreover, in this case we are able to reverse the action of a noise channel and broaden the pseudo-telepathy region. In the case of high values of the noise parameter 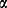, results for the amplitude damping channel resemble those obtained for depolarising channel, as the probability of winning stabilizes around 0.65 for 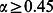 instead of decreasing to approximately 0.5.

## Conclusions

The principal result of this paper is a methodology of partial denoising with the usage of local quantum channels. The presented tool can be used in the cases in which

- the parameters of the noise are accessible,
- the noisy channel is separable and acts independently on each qubit,
- the entangled quantum state the parties use in known in advance,
- the parties have access to quantum computers but
- are no allowed to communicate.

We have proposed a method to reverse an action of a quantum channel using semidefinite programming. The method allows us to find a product channel which partially reverses a given channel. We use the following scheme to achieve this goal. First, we fix all parts of the product, except for one, which is being optimized. After the SDP optimization, we move on to optimize the next part of the product channel, using the value obtained in the earlier step. We repeat this for all parts of the product channel. We run the process a great number of times to obtain a converging solution.

**Figure 8:**
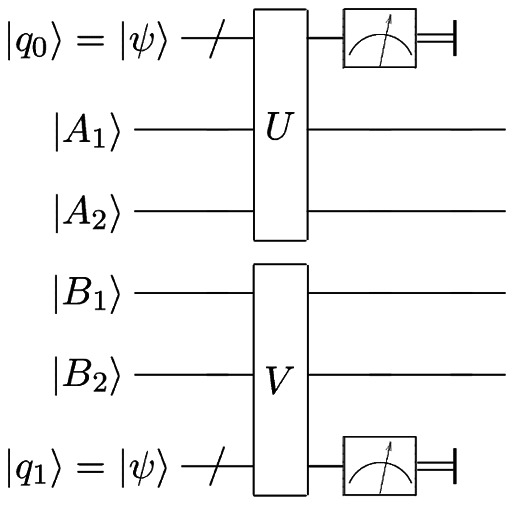
Quantum circuit. A quantum circuit showing the implementation of our scheme. A*_i_* and B*_i_* denote Alice's and Bob's qubits. *q*_0_ and *q*_1_ are the ancillary qubits they need to add.

Obtained channel may be implemented on a real physical system using the Stinespring representation. An example of the quantum circuit implementing this scheme is shown in Fig. 8. Alice and Bob each add ancillary qubits to their original ones. Then they apply a unitary operator on their respective systems. Finally, they perform a measurement on the ancillary qubits, leaving their starting qubits in a less noisy state.

As an example of usage of this optimization scheme we present the quantum pseudo-telepathy magic square game. We obtained results showing an improvement in the players' success rate in the game. Specifically, we were to broaden the range of the noise parameter 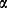 for which the quantum effect occurs.
